# Prebiotic administration normalizes lipopolysaccharide (LPS)-induced anxiety and cortical 5-HT2A receptor and IL1-β levels in male mice

**DOI:** 10.1016/j.bbi.2015.10.007

**Published:** 2016-02

**Authors:** Helene M. Savignac, Yvonne Couch, Michael Stratford, David M. Bannerman, George Tzortzis, Daniel C. Anthony, Philip W.J. Burnet

**Affiliations:** a Clasado Research Services Ltd, Reading RG6 6BZ, UK; b Department of Pharmacology, University of Oxford, Oxford OX1, UK; c CR-UK/MRC Oxford Institute for Radiation Oncology, University of Oxford, OX3 7DQ, UK; d Department of Experimental Psychology, University of Oxford, Oxford OX1, UK; e Department of Psychiatry, University of Oxford, Oxford OX3 7JX, UK

**Keywords:** Inflammation, Behaviour, Frontal cortex, Cytokines, 5-HT2A receptor, NMDA receptor, Microbiota, BGOS

## Abstract

The manipulation of the enteric microbiota with specific prebiotics and probiotics, has been shown to reduce the host’s inflammatory response, alter brain chemistry, and modulate anxiety behaviour in both rodents and humans. However, the neuro-immune and behavioural effects of prebiotics on sickness behaviour have not been explored. Here, adult male CD1 mice were fed with a specific mix of non-digestible galacto-oligosaccharides (Bimuno®, BGOS) for 3 weeks, before receiving a single injection of lipopolysaccharide (LPS), which induces sickness behaviour and anxiety. Locomotor and marble burying activities were assessed 4 h after LPS injection, and after 24 h, anxiety in the light–dark box was assessed. Cytokine expression, and key components of the serotonergic (5-Hydroxytryptamine, 5-HT) and glutamatergic system were evaluated in the frontal cortex to determine the impact of BGOS administration at a molecular level. BGOS-fed mice were less anxious in the light–dark box compared to controls 24 h after the LPS injection. Elevated cortical IL-1β concentrations in control mice 28 h after LPS were not observed in BGOS-fed animals. This significant BGOS × LPS interaction was also observed for 5HT2A receptors, but not for 5HT1A receptors, 5HT, 5HIAA, NMDA receptor subunits, or other cytokines. The intake of BGOS did not influence LPS-mediated reductions in marble burying behaviour, and its effect on locomotor activity was equivocal. Together, our data show that the prebiotic BGOS has an anxiolytic effect, which may be related to the modulation of cortical IL-1β and 5-HT2A receptor expression. Our data suggest a potential role for prebiotics in the treatment of neuropsychiatric disorders where anxiety and neuroinflammation are prominent clinical features.

## Highlights

• The ingestion of BGOS attenuated post-inflammatory anxiety in mice.
• BGOS prevented the LPS-mediated increase in cortical IL-1β levels.
• BGOS prevented the LPS-mediated increase in cortical 5-HT2A receptor levels.

## 1 Introduction

A functional link between the intestinal microbiota and neuronal function in central nervous system has been convincingly established in recent years, which appears to stem notably from alterations in the host immune response (Rhee et al., 2009, Cryan and Dinan, 2012, Burnet and Cowen, 2013, Forsythe and Kunze, 2013). Germ-free mice display a reduction in the ability to clear pathogens (Fritz et al., 2011), an exaggerated hypothalamic–pituitary–adrenal (HPA) reaction to stress (Sudo et al., 2004) and altered anxiety behaviour (Bercik et al., 2010, Neufeld et al., 2011, Diaz Heijtz et al., 2011). Furthermore, the brains of germ-free animals contain microglia that appear less mature than those of specific pathogen free (SPF) mice and the transcriptome of these microglia also suggests that they are less mature (Erny et al., 2015). By contrast, in a strain dependent manner the administration of specific *Bifidobacteria* and/or *Lactobacilli* probiotic species, induce anxiolytic and antidepressant-like actions in rodents and humans (Messaoudi et al., 2011, Dinan et al., 2013, Savignac et al., 2014) by seemingly altering key neurotrophic molecules or neurotransmitter systems involved in anxiety behaviours (Bercik et al., 2010, Bravo et al., 2011, O’Sullivan et al., 2011). Specific probiotic strains also inhibit stress-induced elevations of plasma corticosterone (Gareau et al., 2007, Bravo et al., 2011, Messaoudi et al., 2011) and a wider range of probiotics have been shown to have anti-inflammatory actions (Konieczna et al., 2012).

Prebiotics, which are dietary fibres that promote the proliferation of specific intrinsic *Bifidobacteria* and *Lactobacilli*, may also display similar properties as specific products have been shown to modulate the immune system and display anti-inflammatory properties (Hardy et al., 2013, Kanauchi et al., 2013, Vulevic et al., 2013). Importantly, we have recently demonstrated that fructo-oligosaccharides (FOS) and a specific non-digestible galacto-oligosaccharide formulation (Bimuno®, BGOS), elevate the levels of brain-derived neurotrophic factor (BDNF), as well as central N-methyl-D-aspartate receptor (NMDAR) subunit expression (Savignac et al., 2013). This study also demonstrated the prebiotic properties of BGOS, by showing a significant growth of *Bifidobacteria* after its administration. Furthermore, we have demonstrated that BGOS intake reduces the cortisol awakening response, and some emotional processes in healthy volunteers (Schmidt et al., 2015). These findings, therefore, suggest that certain prebiotics, and in particular, BGOS, may have anxiolytic effects, though this, together with potential underlying mechanisms of action, have not been explored.

Specific pro- and prebiotics have been shown to reduce the levels of circulating pro-inflammatory cytokines such as interleukin-1 (IL-1), IL-6 or tumour necrosis factor alpha (TNF-α) (Hardy et al., 2013, Vulevic et al., 2013). Elevated levels of these cytokines are associated with certain psychiatric disorders (Rook et al., 2014, Stuart et al., 2015, Wohleb et al., 2015). Thus, these findings suggest that the immuno-modulatory action of certain pro- and prebiotics may be key to attenuating inflammation-related aberrant behaviour. Notably, rodents administered with a single, peripheral dose of lipopolysaccharides (LPS) from Gram-negative bacteria, display ‘sickness behaviour’ where a reduction of locomotor activity within the first few hours is a principal feature (Cunningham et al., 2009, Biesmans et al., 2013). This effect is followed by longer term depressive-like behaviour and anxiety. At 24 h post injection mice exhibit no overt changes in locomotor behaviour, but do show increased immobility in a forced swim test, as well as decreased sucrose preference and reduced marble burying activity, indicating a depression-like state (Couch et al., 2015). This time point is suitable for studying the impact of prebiotics as it minimises the confounds associated with the acute reductions in locomotor activity. Attenuation of this LPS-induced sickness behaviour is associated with the normalization of exaggerated IL-1, IL-6 or TNF production, which can be achieved through dietary interventions (e.g. calorific restriction prior to LPS injections (MacDonald et al., 2014)). Since exaggerated immune function has been associated with several neuropsychiatric disorders that are linked to altered glutamate and 5-HT neurotransmission (Mitchell and Dinan, 2010, Marsden, 2011, Dantzer, 2012), the ability to use prebiotics to modify neural function by manipulating the gut microbiota, or via their possible direct effect on the host gut mucosa, in a low cost and physiologically safe manner, is an attractive proposition for the development of new therapy.

Here we tested the potential of BGOS to prevent, or attenuate, LPS-induced sickness and anxiety behaviour in mice. To provide further insights into the mechanisms underlying prebiotic action, we also measured the effect of BGOS on the concentration of key cytokines in the frontal cortex and corticosterone in the plasma, and evaluated the impact of the prebiotic on the cortical glutamatergic and serotonergic systems. The frontal cortex was chosen as a region of interest for its susceptibility to inflammatory neuropathology (de Pablos et al., 2006), notably following LPS administration, and for its role in many neuropsychiatric disorders (Shelton et al., 2011, Couch et al., 2013a, Couch et al., 2013b, Pan et al., 2014).

## 2 Material and methods

### 2.1 Animals

All experiments were carried out with local ethical approval and a UK Home Office licence granted under the Animals (Scientific Procedures) Act (1986). Male CD1 mice (25–30 g, 6–8-week old, Harlan Orlac, UK), were housed 3 per cage (plexiglas cages 33 × 15 × 13 cm, *L* × *W* × *H*) and maintained under standard controlled laboratories conditions (12-h light–dark cycle, lights on at 7 a.m., 21 ± 1 °C, humidity 50 ± 5%). The CD1 mouse is outbred and displays a more exaggerated pro-inflammatory cytokine response to LPS compared to other strains such as the C57Bl6 (Nikodemova and Watters, 2011). Male mice were used to maintain consistency with our previous prebiotic studies in male rats (Savignac et al., 2013), and with previous behavioural studies using probiotics (Bravo et al., 2011, Savignac et al., 2014, Savignac et al., 2015). Social dominance, which may occur in group-housing and interfere with sickness behaviour (Gibb et al., 2011, Wohleb et al., 2012), was thoroughly monitored by trained experimenter (Savignac et al., 2011) but no overt dominance behaviour was observed in the home-cages throughout the experiment. Mice were fed with standard mouse chow *ad libitum* and allowed 4–5 days habituation to the animal facility before receiving prebiotic treatment.

### 2.2 BGOS administration

To minimize stress and reduce the likelihood of fatalities associated with the gavage procedure (Damsch et al., 2011), a pilot study was run to test that the administration of BGOS via the drinking water sufficiently elevated faecal *Bifidobacteria*, and to evaluate an appropriate control solution (see below). The supplementation of drinking water with test compounds is a non-invasive approach which has been previously used to deliver other prebiotics (Bindels et al., 2012) and probiotics (Rashid et al., 2014) to rodents. Since the BGOS mix used in the current study consists of 48% w/w galacto-oligosaccharide, 26% lactose, 14% glucose and 12% galactose, a mixture of these sugars in the same proportion in the absence of galacto-oligosaccharides, was also tested. The enzyme used to covert the lactose to galacto-oligosaccharides is removed by means of filtration (Tzortzis et al., 2005), which circumvents the need to correct for any contaminating protein within the BGOS powder.

Pilot Study: Male mice, housed as described above, were provided with either drinking water containing BGOS, (1.3%, w/v, Bimuno®, Clasado, Reading, UK; *n* = 6), BGOS free sugars (BFS, *n* = 6) or normal drinking water (*n* = 6), in addition to their normal chow, for 3 weeks. The BGOS was freshly prepared daily by dissolving 13 g of BGOS powder in 1L of water. The BFS solution was also freshly prepared by dissolving lactose (3.38 g), glucose (1.82 g) and galactose (1.56 g) (each from Sigma–Aldrich, UK) in 1L of water. Clean standard drinking bottles were then filled with an equal quantity of the solution and provided to all cages. Fluid intake was monitored daily by weighing drinking bottles to ensure all animals were drinking an equivalent quantity of fluid. The prebiotic properties of BGOS have been extensively studied, and the product has been consistently shown to selectively increase *Bifidobacteria* and, to some extent, *Lactobacilli* in both humans and animals (Tzortzis et al., 2005, Depeint et al., 2008, Vulevic et al., 2008, Vulevic et al., 2013, Silk et al., 2009, Savignac et al., 2013). The enumeration of *Bifidobacteria* alone were therefore estimated in our pilot experiment (see below).

### 2.3 Enumeration of faecal Bifidobacteria from pilot study

After the 3 week supplementation with test compounds, all mice were culled and fresh faecal pellets were removed from the large intestine. Total *Bifidobacteria* species were evaluated with Beerens agar plates, a method that has recently been used to enumerate these bacteria in human faecal samples from infants supplemented with galacto-oligosaccharides (Stiverson et al., 2014). Briefly, faecal pellets from each animal were weighed and homogenised in PBS (1:10 w/v) and diluted in a 1:10 series in fresh PBS. An aliquot of 0.1 ml of 3 dilutions each was plated on selective Beerens agar plates in triplicate. They were then incubated at 37 °C for 48 h inside an anaerobic chamber containing anaerobic atmosphere generation bags (Sigma Aldrich, UK). The colonies formed were counted from the plates that had 30–100 colonies. The number of CFU/ml of culture for each treatment was calculated based on the number of colonies and the corresponding dilution factor. The values were then expressed as CFU/g faeces which was calculated from the original weight of the faecal matter sampled.

### 2.4 Experimental design and LPS injections

**Fig. 1:**
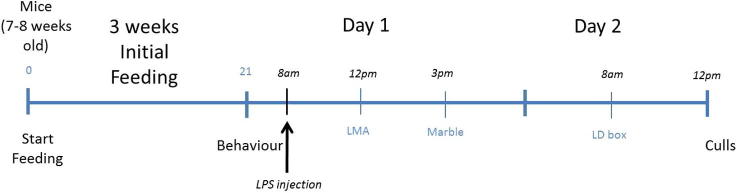
Schematic of the experimental design. CD1 mice received standard chow *ad libitum* with either drinking water alone or water containing BGOS (1.3% w/v), for 3 weeks. Animals then received a single injection of either LPS (0.75 mg/kg) or saline 4 h prior to locomotor activity, which was followed after a period of rest by the marble burying test. The mice were then tested for anxious behaviour 24 h after the LPS or saline injection, in the light/dark box, and then sacrificed 3 h after.

The schematic of the main study is presented in Fig. 1. Based on the data from the pilot study (see Section 3.1), mice were provided with normal drinking water or a BGOS solution with their standard food. After 3 weeks of supplementation, all animals received drinking water 24 h prior to LPS injections, to ‘wash-out’ any residual circulating sugars, particularly glucose, absorbed from the BGOS solution. This was a precautionary measure, although previous studies have shown that the oral administration of 50 mg glucose did not significantly alter blood sugars levels (Andrikopoulos et al., 2008); in the current study, mice ingested an average of 13 mg glucose/day. The galacto-oligosaccharides in BGOS are not absorbed by the intestines, but are readily metabolized by the microbiota in the colon. BGOS has also been suggested to interact directly with gut cells (Tzortzis, 2009). Therefore, the observed effects of BGOS supplementation are likely to arise from either the gut bacteria and/or direct interaction with the gut.

A single injection of 0.75 mg/kg LPS (*E. coli* 026:B6, Sigma–Aldrich) in saline (0.9%), or saline alone, was administered to mice by an intraperitoneal route, 4 h before behavioural testing commenced. Four groups (*n* = 15 mice/group, 5 cages of 3 mice per group) were tested: (1) water-fed/saline injected (Water/SAL); (2) water-fed/LPS injected, (Water/LPS); (3) BGOS-fed/saline injected (BGOS/SAL); and (4) BGOS-fed/LPS injected (BGOS-LPS). This experiment was performed twice to provide a total of 30 mice per test group for analysis.

Mice were tested for locomotor activity 4hrs post LPS/saline injections, followed by the marble burying test 7hrs after the injection. This time-window was carefully chosen to enable behaviour to be assessed at the peak of LPS-induced sickness and pro-inflammatory cytokine release (Dantzer et al., 2008). Twenty-four hours post injection, animals were tested in the light–dark box for anxiety behaviour (Couch et al., 2015). The experimenter was blind to treatment and remained in the experimental room silently during testing. Animals were tested in a counter-balanced, pseudo-random order with respect to cage and treatment groups. Animals were culled 3 h post behaviour to assess the effects of LPS on brain chemistry.

### 2.5 Locomotor activity (LMA)

The administration of LPS reduces LMA (Skelly et al., 2013), and is often used as one measure of sickness behaviour. The test was run in a transparent plexiglas box (48 × 27 × 21 cm, *L* × *W* × *H*, Photo Beam Activity Hardware and Software, Open Field San Diego Instruments) covered with a transparent plexiglas top (perforated for breathing) and containing a thin layer of sawdust bedding. Room lighting was 60 ±5 lux. Four hours following LPS or saline injections, each animal was gently placed individually into the locomotor activity box and allowed to explore freely for 2 h. LMA was recorded using photo-beams arranged across the box and is expressed as the number of beam breaks made by the animals per unit time (10 min bins). At the end of the test animals were returned to their home cage to rest before the next behavioural test. The LMA box was cleaned between animals with 70% ethanol to remove odours.

### 2.6 Marble burying

Spontaneous digging by CD1 mice has been shown to be impaired in IL-1β-mediated sickness behaviour (Cirulli et al., 1998). A concomitant change in the cortical serotonin system has also been shown with post-inflammatory impairment of digging behaviour (Couch et al., 2015). We therefore applied the marble burying paradigm to assess the effects of acute inflammation on digging activity in mice, and the influence of BGOS supplementation thereon. The test was conducted 7hrs following LPS/saline injection, as previously described (Njung’e and Handley, 1991, Deacon, 2006). Twenty marbles were placed on top of 5-cm sawdust bedding in transparent plastic cages (44 × 28 × 12 cm, *L* × *W* × *H*), in 5 lines of 4, 2 cm away from each other and 2 cm away from the edges of the cage. Testing occurred under normal room lighting, (∼100 lux at 1 m above the floor), as previously described (Deacon, 2006). Each animal was gently placed in the cage with the marbles for 30 min, after which the number of marbles buried to at least 2/3 of their surface was counted.

### 2.7 Light–dark box

This test was used to assess anxiety behaviour and is based on the approach/avoidance conflict mice face between their attraction for novelty and their fear for bright open arenas. This task has also been shown to be sensitive to behaviours related to changes in the serotonin system and cytokine concentrations in the frontal cortex (Couch et al., 2013a). The apparatus consisted of a small black covered compartment (21 × 16 × 16 cm, *L* × *W* × *H*, with a small opening for access to the light part, 3 × 2.7 cm, *W* × *H*) and a brighter open compartment (46.5 × 21 × 21 cm, *L* × *W* × *H*). Testing was performed under a slightly dim light (50 lux within the bright compartment), as previously described (Strekalova et al., 2004). Twenty-four hours following LPS or saline injections, each animal was gently placed in the dark part of the light–dark box and was free to explore the whole box for 5 min. The latency to leave the dark area, number of transitions between the dark and lights parts and the time spent in the light section were measured. The criterion to enter any compartment was 4 paws in that compartment. Mice were returned to their home cage with cage mates at the end of the procedure.

### 2.8 Tissue collection

Animals were sacrificed between 12 and 1 p.m., and 3hrs following light/dark box testing. Mice were gently removed from their cages and swiftly culled by concussion and decapitation within 30 s, in accordance with Home Office, UK, guidelines. An experienced experimenter ensured that care was taken to avoid unnecessary distress to the animals. Whole brains were immediately harvested and snap-frozen in cold isopentane on dry-ice (Sigma–Aldrich, UK) before storage at −80 °C until further analysis. Trunk blood was collected in Ethylene Diamine Tetra Acetic Acid (EDTA) tubes and spun for 15 min at 5000 rpm. Plasma was isolated and stored at −80 °C prior to analysis.

### 2.9 Cytokine assays

Each mouse cage (5 cages/group) yielded 3 brains of which one from each cage was randomly selected for analysis. An additional brain from each group was arbitrarily chosen to provide 6 brains per group for investigation. This selection procedure was also carried out with the replicate samples so that 12 brains (6 from each replicate), from each of the 4 groups were processed. Frontal cortex tissue from one hemisphere was removed with a scalpel on dry-ice, weighed and homogenized in RIPA buffer (1:5 w/v, Sigma Aldrich, UK) containing protease inhibitors (‘Complete-Mini’, Roche). Protein concentrations were determined using the Bradford reagent (Sigma, UK). The concentrations of TNF-α, IL-1β, IL-6 and IL-10 in all protein samples were measured using commercial ELISA kits (R&D Systems), following pre-determined dilutions of the extracts in respective assay buffer. All ELISAs were performed according to manufacturer’s recommendations.

### 2.10 Western blotting

Western blots were performed on the cortical extracts used for cytokine assays as previously described (Savignac et al., 2013). Briefly, equal concentrations of protein extracts of cortex (5 μg) from experimental and control groups (*n* = 12 samples/group) were mixed with loading buffer (50 mM 1, 4-dithiothreitol and 0.025% bromophenol blue) and fractionated with a molecular weight marker (GE Healthcare, Buckinghamshire, UK) by electrophoresis on pre-cast 7.5% SDS/polyacrylamide gels (Biorad, UK), and trans-blotted onto polyvinyl difluoride (PVDF) membranes (Immobilon-P, Millipore, Watford, UK).

The membranes were blocked with 5% (w/v) non-fat milk in PBS containing 0.1% Tween^20^ (PBST) for 45 min, and then incubated for 1 h at room temperature in incubation buffer (PBST with 2% [w/v] milk) containing a primary antibody (diluted 1:1000) against one of three NMDAR subunits: NR1 (AB9864, Millipore, UK, diluted 1:1000), NR2A (AB1555, Millipore, UK, diluted 1:2000) and NR2B (AB15362, Millipore, UK, diluted 1:2000); the 5-HT1AR (ab85615, Abcam, UK, diluted 1:2000), the 5-HT2AR (ab85496, Abcam, UK, diluted 1:1000) and β-actin (Sigma–Aldrich, UK, diluted 1:50,000). Membranes were then washed 3 times for 10 min in PBST and incubated for 30 min in HRP-linked secondary antibody in blocking buffer. Immunoreactive bands were visualized by chemiluminescence using the ECL-Plus kit (GE Healthcare, Buckinghamshire, UK) and apposing membranes to X-ray film (Kodak BioMax AR film). All antibodies produced a single band of expected molecular weight. The optical densities (OD) of bands were measured using the AlphaImager 3400, and the data expressed as OD ratios of NMDAR subunit: β-actin.

### 2.11 HPLC analysis

The levels of serotonin (5HT) and 5-hydroxyindole acetic acid (5-HIAA) were measured in frontal cortex tissue dissected from the brain hemisphere contralateral to that used for protein extraction (see above). Thus, a total of 12 samples per group (6 per replicate, chosen as described for protein extraction), were subjected to HPLC analysis as previously described (Nguyen et al., 2010), with some modifications. Fragments of cortical tissue were homogenized in 0.06 M perchloric acid (1:10 w/v) and then centrifuged (14,000*g*, 5 min) to remove any tissue debris. All samples were analysed using HPLC with electrochemical detection and separated with an ACE column (C18, 3 μm, 125 × 3 mm + ACE C18 guard, 10 × 3 mm run at 35 °C). The eluent was as previously described (Nguyen et al., 2010) (9% methanol, 50 mM citric acid, 50 mM H_3_PO_4_, 0.1 mM Na_2_EDTA, 2 mM 1-octanesulphonic acid, pH 3.2) pumped with a flow rate of 0.6 ml/min (Shimadzu LC10 ADVP HPLC Pump). Monoamines were detected using a porous graphitic electrode held at +0.40 V (Coulochem 5100A). The sample content was determined with reference to daily-calibrated standard solutions in 0.06 M perchloric acid (5 pmol 5-HT and 5-HIAA). Chromatograms were displayed and analysed using Shimadzu LC solution software.

### 2.12 Data analysis

All data are expressed as mean ± standard error of the mean, and were analysed using SPSS software (version 19). Statistical outliers (>3 × SD) identified by the software were removed from the analyses. Normal distribution of the data was assessed using the Shapiro–Wilk test. Non-parametric data were subjected to square-root transformation prior to analysis. The LMA data from all four groups were analysed with an ANOVA which comprised between subjects factors of ‘diet’ (water, BGOS) and ‘treatment’ (saline, LPS), and a within-subjects factor (repeated measure) of ‘time’. All other data were analysed in a standard 2-way ANOVA with ‘diet’ and ‘treatment’ as between-subjects factors. *Post hoc* Bonferroni comparisons were made where appropriate. In addition, analysis of simple main effects was also used to explore further the basis of significant interactions from locomotor activity data (Howell, 1997).

## 3 Results

### 3.1 Pilot study on BGOS administration

**Fig. 2:**
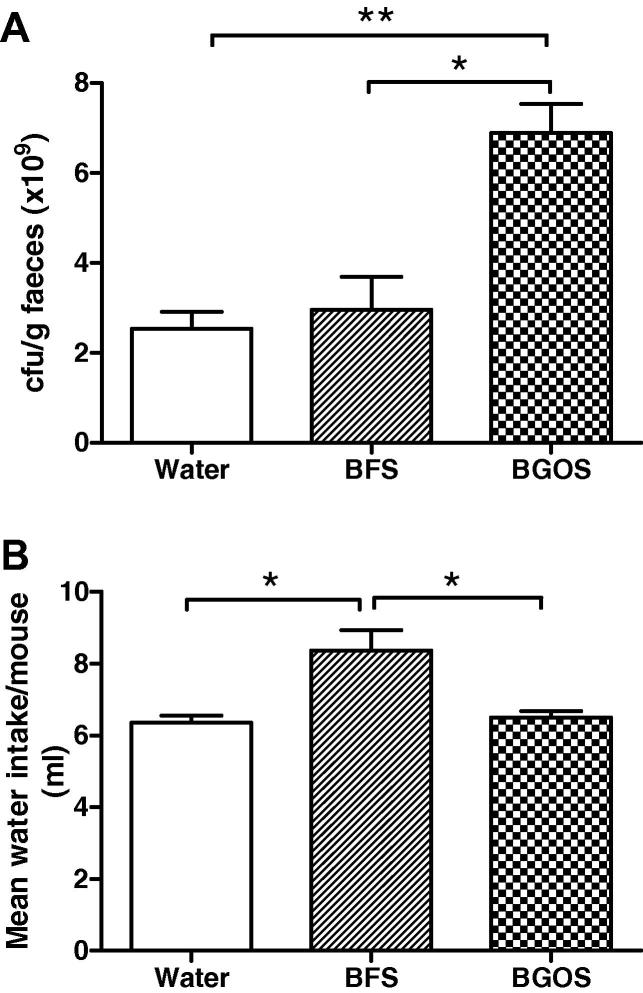
Effect of BGOS and control solutions on faecal *Bifidobacteria* numbers and fluid intake in CD1 mice. (A) There was a significant effect of BGOS on faecal *Bifidobacteria* numbers compared to mice provided with drinking water supplemented with BGOS free sugars (BFS), or water alone. (B) The fluid intake of mice provided with a BFS solution for 3 weeks, was significantly greater that than those provided with BGOS or water alone. Data are presented as mean ± SEM. ^∗^*p* < 0.05, ^∗∗^*p* < 0.01. *n* = 6 mice/group.

The effect of supplementing drinking water with BGOS or BFS compared to normal water on faecal *Bifidobacteria* numbers, is shown in Fig. 2. One-way ANOVA followed by *post hoc* Bonferroni comparisons revealed that there was a significant elevation of *Bifidobacteria* in the BGOS-fed mice compared to BFS-fed (*t* = 4.57, *p* < 0.05) and the water group (*t* = 5.06, *p*, 0.05) (Fig. 2A). There were no statistical differences between the water and BFS-fed groups (*t* = 0.49, *p* > 0.05). However, mice provided with a BFS solution drank significantly more fluid within the 3 week test period than BGOS (*t* = 3.59, *p* < 0.05) or water-fed animals (*t* = 3.86, *p* < 0.05) (Fig. 2B). There was no statistical difference between water and BGOS-fed mice (*t* = 0.27, *p* > 0.05). On the basis of these findings, therefore, normal drinking water was used as a control in the main experiment.

### 3.2 Effects of LPS injection and BGOS intake on LMA and marble burying behaviour in mice

**Fig. 3:**
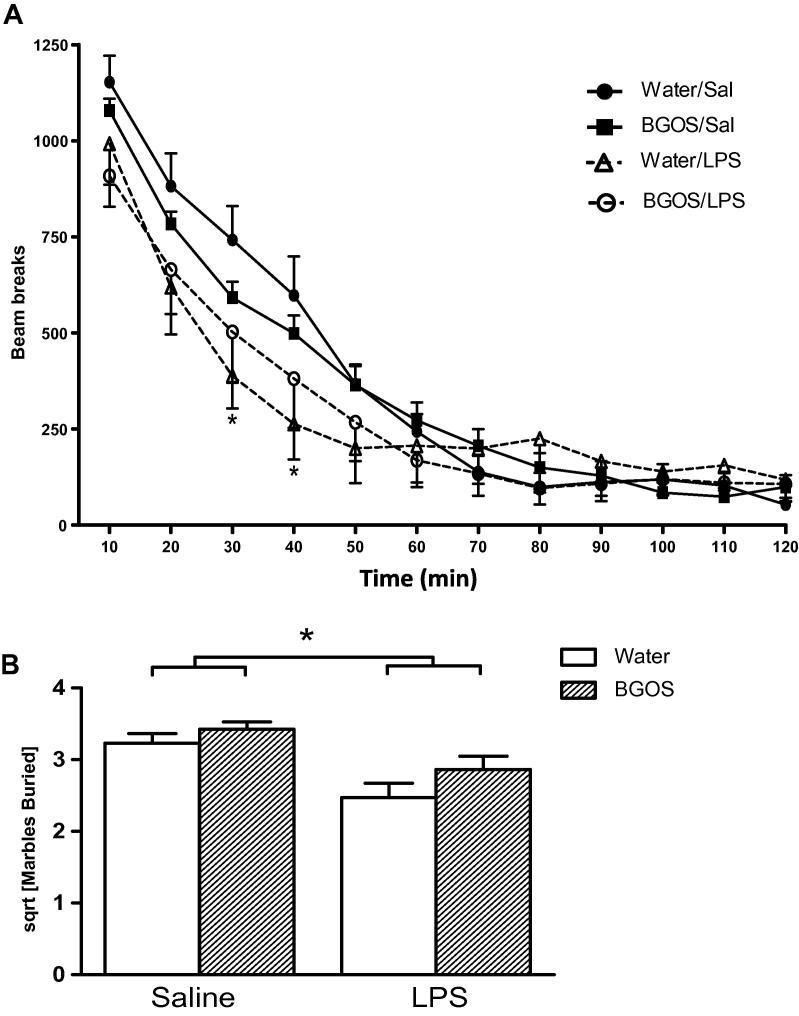
Effects of BGOS supplementation on locomotor activity and marble burying after LPS or saline injections. (A) Locomotor activity of all mice progressively decreased over the 2 h exploration of the cage. There was a main effect of LPS in water-fed mice relative to their saline administered counter-parts (^∗^*p* < 0.05). However, there were no significant differences between Water/LPS and BGOS/LPS. (B) The number of marbles buried by mice were square-root (sqrt) transformed. A reduction in marble burying was observed in LPS treated mice compared to saline administered animals (indicated by bars). Data are presented as mean ± SEM. ^∗^*p* < 0.05. (A) *n* = 27 mice/group. (B) *n* = 28 mice/group for Water/SAL, Water/LPS and BGOS/SAL; *n* = 27 mice/group for BGOS/LPS.

The results of the 2 h LMA test are shown in Fig. 3A. LPS treatment reduced locomotor activity compared to saline injected mice. BGOS pre-treatment influenced the locomotor activity data but these effects were complex and somewhat difficult to interpret. An ANOVA with between subject factors of diet (water vs. BGOS) and treatment (saline vs. LPS), and a within-subjects factor of time (12 time bins of 10 min each), revealed a significant three-way diet × treatment × time bin interaction (*F*_11,1144_ = 2.75; *p* < 0.005), and a significant treatment × time bin interaction (*F*_11,1144_ = 6.75; *p* < 0.0001), which reflected the fact that LPS treatment reduced locomotor activity levels relative to saline treatment between 20 and 40 min post-injection (analysis of simple main effects; *F*_1,104_ > 3.97; *p* < 0.05 for time bins 20, 30 and 40 mins). There was neither a significant diet × treatment interaction (*F*_1,104_ = 0.003; *p* = 0.955), nor an overall effect of diet (*F*_1,104_ = 0.01; *p* = 0.922), nor a main effect of treatment (*F*_1,104_ = 1.09; *p* = 0.3).

To investigate further the basis for this significant 3 way interaction between diet, treatment and time, we ran separate two way ANOVAs to investigate specifically the effects of each diet and of each treatment. A two-way ANOVA for the animals maintained on a water diet (with LPS vs. saline treatment as a between subjects factor, and time bin as a within-subjects factor) revealed a significant interaction between LPS treatment and time bin (*F*_11,572_ = 8.80; *p* < 0.0001) which was due to reduced locomotor activity in the LPS treated mice at 30 and 40mins (simple main effects; *F*_1,52_ > 5.94; *p* < 0.02). There was no overall main effect of LPS treatment (*F*_1,52_ < 1; *p* > 0.50). In contrast, a two-way ANOVA of LMA data from mice on the BGOS diet failed to reveal a significant main effect of LPS treatment, or an LPS treatment by time bin interaction (*F*_11,572_ < 1; *p* > 0.30). This would appear to suggest that LPS treatment had no effect in BGOS fed mice. However, a separate two way ANOVA comparing the effect of LPS treatment in water and BGOS fed animals failed to reveal a significant difference between these groups. There was no main effect of diet (BGOS vs. water; F < 1; *p* > 0.93), and no interaction between diet and time bin (F _11,572_ = 1.13; *p* > 0.30) for LPS treated mice. Notably, a two way ANOVA comparing BGOS and water diet for the saline injected animals did reveal a significant diet by time bin interaction (*F*_11,572_ = 2.57; *p* < 0.005), which was due to higher activity levels in BGOS/SAL mice compared to Water/SAL mice at 70 and 80min post-injection (simple main effects; *F*_1,52_ > 4.82; *p* < 0.05).

Overall, these data did not convincingly demonstrate an influence of BGOS on the reduction of LMA following an LPS injection. The decrease in LMA in water-fed mice following LPS was mild and only significant 30 and 40 min after the endotoxin administration, relative to saline injected mice. Although this was not observed in BGOS mice injected with LPS, the lack of a significant difference between the BGOS/LPS and Water/LPS animals at these and any other time points, precludes unequivocal conclusions to be drawn.

In the marble burying test (Fig. 3B), there was no significant diet × treatment interaction (*F*_1,107_ = 0.71, *p* = 0.40), though LPS treatment alone did have a significant effect on marble burying (*F*_1,107_ = 8.46, *p* = 0.004).

### 3.3 Effects of LPS injection and BGOS intake on anxiety behaviour in the light–dark box

**Fig. 4:**
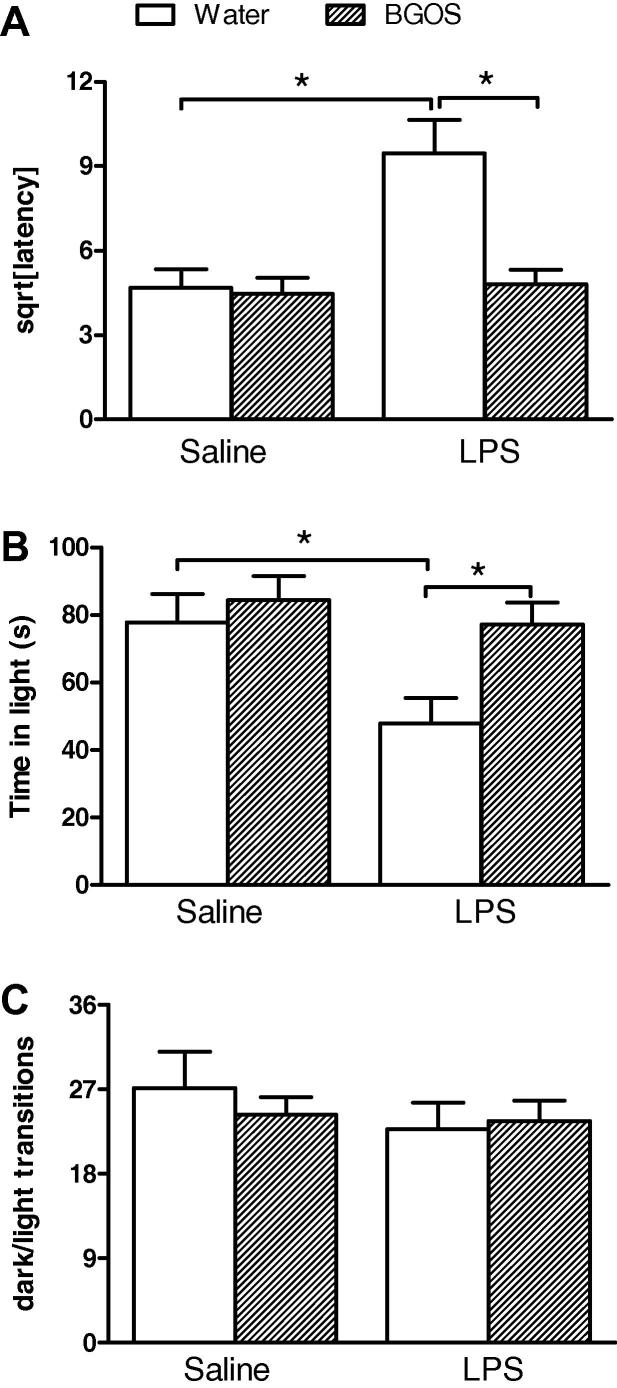
BGOS supplementation attenuates LPS-induced anxiety in the light–dark box. (A) Mice that had received normal drinking water, demonstrated an increased latency to enter the lit area 24 h after an LPS injection, compared to BGOS supplemented animals. There was a significant diet × treatment interaction. Bars indicate significance between group differences after *post hoc* (Bonferroni) analysis. (B) Mice that had received BGOS in their drinking water, spent more time in the lit area compared to controls, 24 h after an LPS injection. There was a significant diet × treatment interaction. Bars indicate significant group differences after *post hoc* (Bonferroni) analysis. (C) LPS injection and BGOS supplementation did not affect the number of transitions between the light and dark areas of the test apparatus. Data are presented as mean ± SEM. All non-parametric data were square-root (sqrt) transformed. ^∗^*p* < 0.05. *n* = 28 mice/group for Water/SAL, Water/LPS and BGOS/SAL; *n* = 27 mice/group for BGOS/LPS.

The influence of LPS and BGOS on mouse behaviour in the light–dark box is shown in Fig. 4.

Latency (Fig. 4A): There was a significant diet × treatment interaction for latency to leave the dark compartment (*F*_1,107_ = 9.44, *p* = 0.003), with significant effects of diet (*F*_1,107_ = 10.62, *p* = 0.0015) and treatment (*F*_1,107_ = 8.70, *p* = 0.004) alone. *Post-hoc* Bonferroni comparisons revealed that there were significant differences between the Water/LPS vs BGOS/LPS animals, and the Water/SAL vs Water/LPS groups (*p* < 0.001), but not BGOS/SAL vs BGOS/LPS or Water/SAL vs BGOS/SAL (*p* > 0.05).

Time in light (Fig. 4B): A similar pattern of results was obtained for time spent in the light section. There was a significant diet × treatment interaction for the time mice spent in the light compartment (*F*_1,107_ = 4.34, *p* = 0.0396), with significant effects of diet (*F*_1,107_ = 11.25, *p* = 0.0011) and treatment (*F*_1,107_ = 6.94, *p* = 0.01) alone. *Post-hoc* Bonferroni comparisons revealed that there were significant differences between the Water/LPS vs BGOS/LPS animals, and the Water/SAL vs Water/LPS groups (*p* < 0.01), but not BGOS/SAL vs BGOS/LPS or Water/SAL vs BGOS/SAL (*p* > 0.05).

Transitions (Fig. 4C): There was no significant diet × treatment interaction for the total number of crossings (transitions) between the light and dark compartments (*F*_1,107_ = 0.83, *p* = 0.36). There was neither a main effect of diet (*F*_1,107_ = 0.29, *p* = 0.59), nor a main effect of treatment (*F*_1,107_ = 2.26, *p* = 0.14) for this total number of transitions.

### 3.4 Effects of LPS injection and BGOS intake on the levels of cytokines

**Fig. 5:**
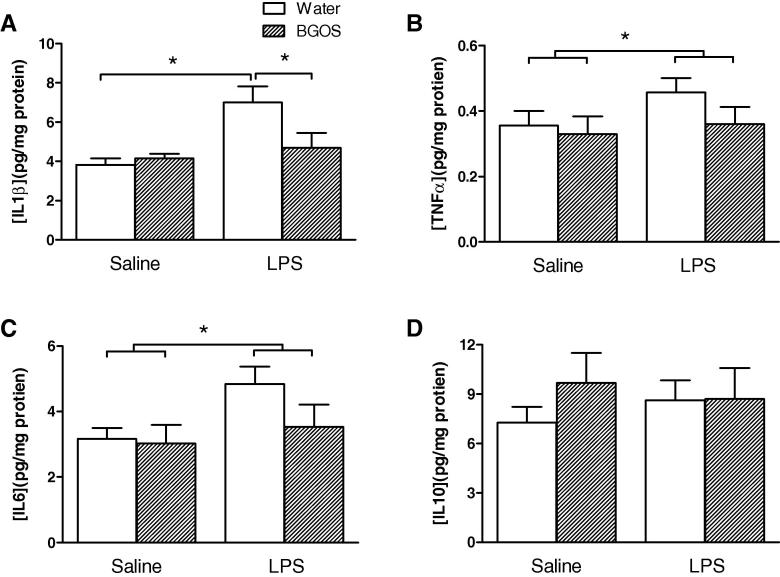
Effect of BGOS supplementation on cytokine levels in the frontal cortex, 28 h after LPS or saline injections. (A) There was a significant diet × treatment interaction, and cortical IL-1β levels in Water/LPS mice were significantly greater than in BGOS/LPS and Water/Sal animals. Bars indicate significant group differences after *post hoc* (Bonferroni) analysis. (B) There was no diet × treatment interaction for TNF *α* levels, though a main effect of BGOS (not indicated) and LPS (indicated by bars) alone were observed. (C) There were neither a diet × treatment interaction nor a main effect of BGOS for IL-6 concentrations, but an effect of LPS was observed (indicated by bars). (D) There were neither a diet × treatment interaction nor main effects of BGOS or LPS on IL-10 concentrations. Data are presented as mean ± SEM. ^∗^*p* < 0.05. *n* = 12 mice/group for Water/SAL and BGOS/SAL; *n* = 11 mice/group for Water/LPS and BGOS/LPS.

The influence of LPS and BGOS on frontal cortex cytokine concentrations are shown in Fig. 5. For the levels of IL-1β (Fig. 5A) there was a significant diet × treatment interaction (*F*_1,42_ = 4.946, *p* = 0.035), with a significant effect of treatment (*F*_1,42_ = 9.365, *p* = 0.005), but not diet (*F*_1,42_ = 2.821, *p* = 0.11). *Post-hoc* Bonferroni comparisons revealed that there were significant differences between the Water/LPS vs BGOS/LPS animals, and the Water/SAL vs Water/LPS groups (*p* < 0.05), but not BGOS/SAL vs BGOS/LPS or Water/SAL vs BGOS/SAL (*p* > 0.05).

There was no significant diet × treatment interaction for TNF α levels, (*F*_1,42_ = 3.473, *p* = 0.073), though significant effects of diet (*F*_1,42_ = 5.604, *p* = 0.025) and treatment (*F*_1,42_ = 4.428, *p* = 0.045) alone were observed (Fig. 5B). There was neither a diet × treatment interaction (*F*_1,42_ = 2.33, *p* = 0.15), nor an effect of diet alone (*F*_1,42_ = 1.03, *p* = 0.32) on the levels of brain IL-6 (Fig. 5C), though a significant effect of LPS was observed (*F*_1,42_ = 5.878, *p* = 0.022). Finally, there was neither a diet × treatment interaction (*F*_1,42_ = 2.33, *p* = 0.15), nor an effect of diet (*F*_1,42_ = 1.03, *p* = 0.32) nor treatment (*F*_1,42_ = 1.03, *p* = 0.32) on the levels of brain IL-10 (Fig. 5D).

### 3.5 Effects of LPS injection and BGOS intake on the levels of 5-HT receptors and NMDAR subunits

**Fig. 6:**
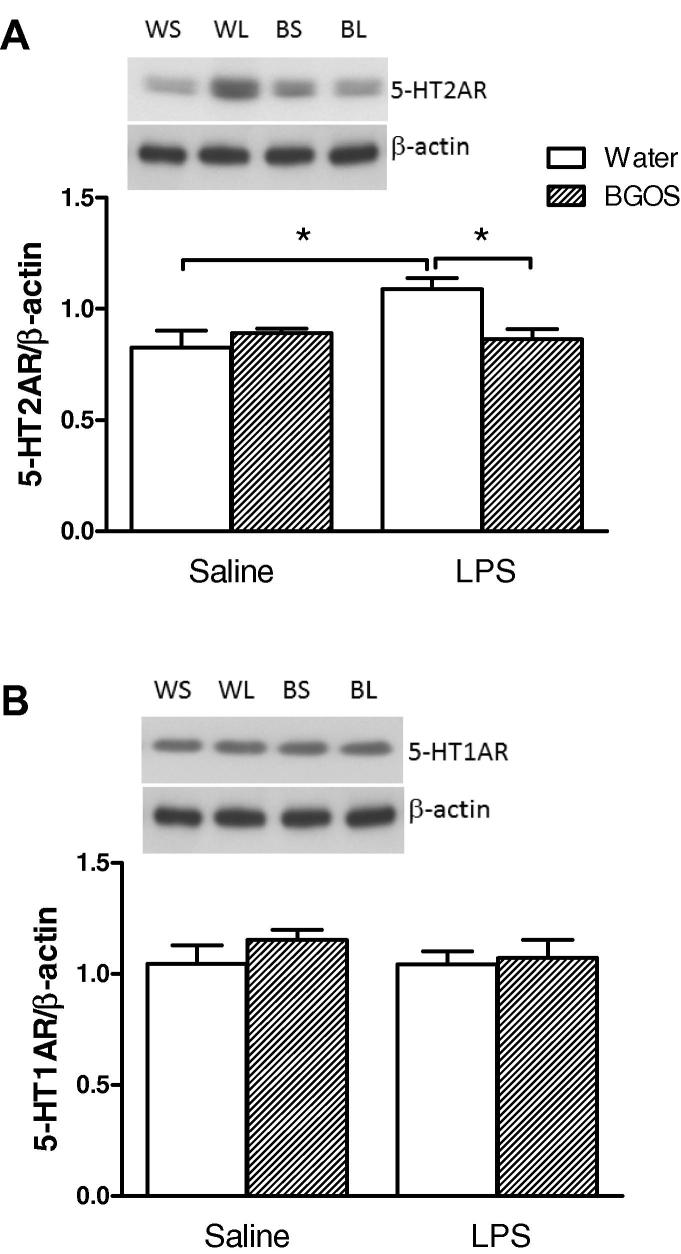
Effects of BGOS supplementation on the 5-HT2AR and 5-HT1AR in the frontal cortex, 28 h after LPS or saline injections. (A) Representative western blot images of 5-HT2ARs in each group (top panel), and receptor:β-actin ratios, following densitometry of western blot images (bottom panel). There was a significant diet × treatment interaction, and cortical 5-HT2AR levels in Water/LPS mice were significantly greater than in BGOS/LPS and Water/Sal animals. Bars indicate significant group differences after *post hoc* (Bonferroni) analysis. (B) Western blot images and 5-HT1AR:β-actin ratios in the frontal cortex. There were neither a diet × treatment interaction nor main effects of BGOS or LPS on 5-HT1AR concentrations. Data are presented as mean ± SEM. ^∗^*p* < 0.05. *n* = 12 mice/group for Water/SAL (WS in top panel) and BGOS/SAL (BS); *n* = 11 mice/group for Water/LPS (WL) and BGOS/LPS (BL).

**Fig. 7:**
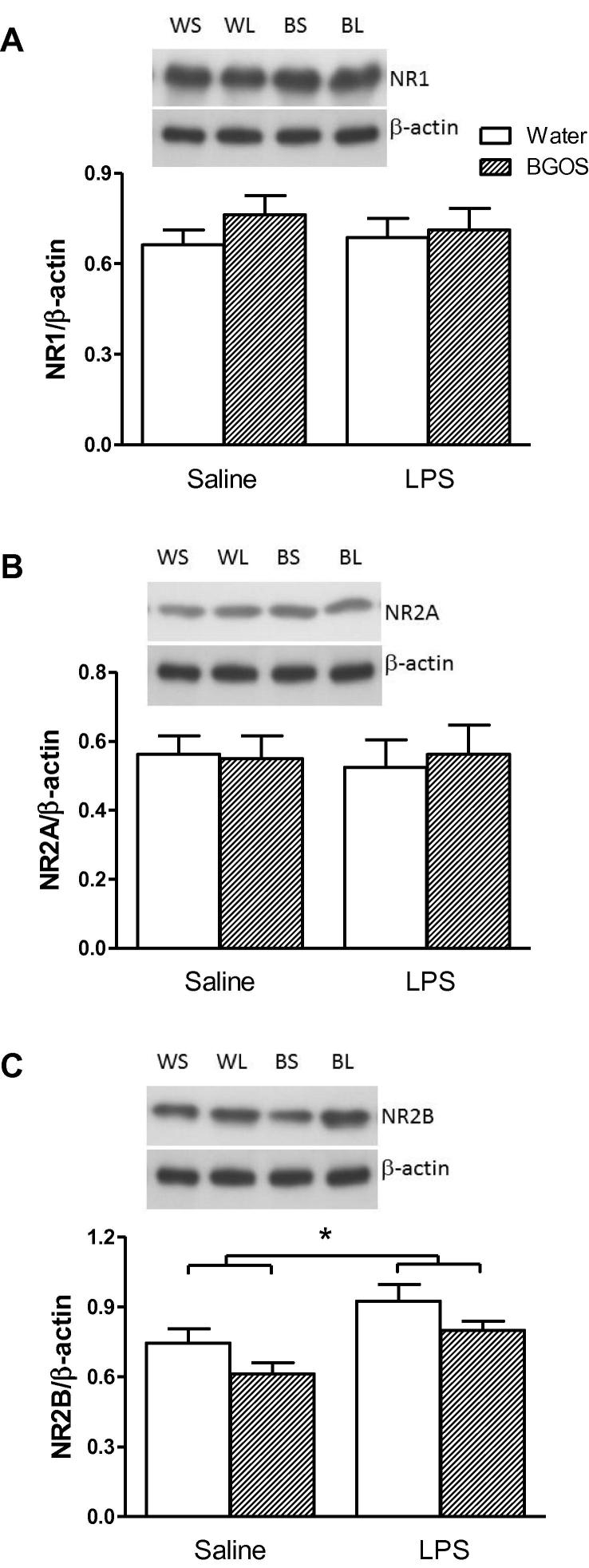
Effects of BGOS supplementation on NMDAR subunits in the frontal cortex 28 h after LPS or saline injections. (A) Representative western blot images of NR1 subunits in each group (top panel), and NR1:β-actin ratios, following densitometry of western blot images (bottom panel). There were neither a diet × treatment interaction nor main effects of BGOS or LPS on NR1 levels. (B) Western blot images and NR2A:β-actin ratios in the frontal cortex. There were neither a diet × treatment interaction nor main effects of BGOS or LPS on NR2A concentrations. (C) Western blot images and NR2B:β-actin ratios in the frontal cortex. Main effects of BGOS (not indicated) and LPS (indicated by bars) alone were observed. Data are presented as mean ± SEM. ^∗^*p* < 0.05. *n* = 12 mice/group for Water/SAL (WS in top panel) and BGOS/SAL (BS); *n* = 11 mice/group for Water/LPS (WL) and BGOS/LPS (BL).

The effects of LPS and BGOS feeding on cortical 5-HT1AR and 5-HT2AR, and NMDAR subunits are shown in Fig. 6, Fig. 7 respectively. There was a significant diet × treatment interaction for 5-HT2AR (*F*_1,42_ = 7.142, *p* = 0.013), with an effect of treatment (*F*_1,42_ = 4.419, *p* = 0.046), but not diet (*F*_1,42_ = 1.409, *p* = 0.21). *Post-hoc* Bonferroni comparisons revealed that there were significant differences between the Water/LPS vs BGOS/LPS animals, and the Water/SAL vs Water/LPS groups (*p* < 0.05), but not BGOS/SAL vs BGOS/LPS or Water/SAL vs BGOS/SAL (*p* > 0.05). There was no significant diet × treatment interaction for 5-HT1AR (*F*_1,43_ = 0.364, *p* = 0.55), and no significant main effects of diet (*F*_1,42_ = 0.98, *p* > 0.05) or treatment (*F*_1,42_ = 0.394, *p* > 0.05) .

The analysis of NMDAR subunits revealed that there were no significant diet × treatment interactions for NR1 (*F*_1,42_ = 0.487, *p* = 0.41), NR2A (*F*_1,42_ = 0.638, *p* = 0.39) or NR2B (*F*_1,42_ = 1.37, *p* = 0.25). However, NR2B levels were affected by treatment (*F*_1,42_ = 7.04, *p* = 0.014) and diet (*F*_1,42_ = 5.25, *p* = 0.031) alone.

### 3.6 Effects of LPS injection and BGOS intake on cortical 5-HT and 5-HIAA levels

The cortical concentrations of 5-HT and 5-HIAA, and 5-HIAA/5-HT ratios are shown in Table 1. There were neither a diet × treatment interaction nor main effects of diet or treatment on cortical 5-HT (*F*_1,42_ = 0.002, *p* = 0.97; diet: *F*_1,42_ = 0.451, *p* > 0.05; treatment: *F*_1,42_ = 2.666, *p* > 0.05), 5-HIAA (*F*_1,42_ = 0.211, *p* = 0.65; diet: *F*_1,42_ = 2.031, *p* > 0.05; treatment: *F*_1,42_ = 0.032, *p* > 0.05) or 5-HIAA/5-HT ratios (*F*_1,42_ = 0.055, *p* = 0.82; diet: *F*_1,42_ = 0.037, *p* > 0.05; treatment: *F*_1,42_ = 1.821, *p* > 0.05).

## 4 Discussion

The current study examined the influence of the prebiotic BGOS on LPS-induced sickness behaviour, anxiety and cytokine expression in mice. Our rationale was based on increasing evidence that the gut microbiota plays an important role in the modulation of immunity, and as such, in the regulation of behaviour. Here we show that BGOS supplementation in mice attenuated post-inflammation anxiety after a single injection of LPS, compared to controls, as well as diminishing the LPS-mediated elevation of IL1-β and 5-HT2ARs in the frontal cortex. Overall, these data support the contemporary theory that the intestinal microbiota are involved in the maintenance of normal brain function, and confirms the notion that regulation of the immune system may be integral to this action.

Post-inflammation anxiety in rodents is a well-known concept. Bassi et al. (2012) have recently demonstrated that LPS injected rats exhibit anxious behaviour in the light–dark box test, which is consistent with our present study using mice. Our key finding, that BGOS feeding prevented LPS-induced anxiety in the light–dark box, is consonant with the action of anxiolytic and antidepressant drugs in the same paradigm (Ohgi et al., 2013). Furthermore, our data are in-keeping with a recent study showing that the oral administration of specific probiotics prior to the induction of liver inflammation, prevented deficits in social exploratory behaviour, a feature of sickness behaviour (Wohleb et al., 2012). However, our study is of particular significance because we show for the first time the beneficial effects on behaviour following the proliferation of *indigenous* gut bacteria, and that this was achieved with a natural, highly stable dietary compound.

The BGOS prebiotic mixture used in the current study contains galacto-oligosaccharides that are resilient to temperature changes and the harsh environment of the gastrointestinal tract. From our data it is clear that the BGOS-mediated bacterial growth is sufficient to impart robust immune and central effects. In contrast, probiotics are more fastidious and very sensitive to environmental changes, and so the number of viable microorganisms reaching the large intestine following their ingestion can vary (Gueimonde and Sánchez, 2012). Moreover, probiotics only promote the growth of one or several beneficial bacteria, whereas BGOS augments the proliferation of all (>30) *Bifidobacteria* species, and some *Lactobacilli*, and is therefore likely to impart stronger influence on neuro-immune interactions.

All microbes possess microbe-associated molecular patterns (MAMPs, formally known as pathogen-associated molecular patterns, PAMPs [Mackey and McFall, 2006]) which can be molecular components of their cell wall (such as LPS on *E. coli*), bacterial flagella, and/or microbial nucleic acids. The activation of pattern recognition receptors by MAMPs initiates the innate immune response which, in the case of the beneficial gut bacteria, may result in the secretion of anti-inflammatory cytokines (Chu and Mazmanian, 2013) Although the mechanisms are uncertain, it is possible that the *Bifidobacteria*, and indeed other commensals, express MAMPs that do not fully activate the host enteric toll-like receptors (TLRs), such as TLR4, that mediate a pro-inflammatory response, but have sufficient affinity for the receptor to allow it to compete with pathogenic MAMPs such as LPS. Clearly, the commensal microbiota significantly contribute to the homeostasis of host immunity, but the mechanisms by which this influence is communicated to the brain are still unclear.

Microglia are the resident macrophages of the CNS and their activation results in the increase of pro- and anti-inflammatory cytokines. A systemic injection of LPS, leads to the elevation of circulating pro-inflammatory cytokines, such as Il-1β and TNF α, which trigger the local production of cytokines in the CNS by stimulating the microglia (Prinz et al., 1999). Thus, if the peripheral innate immune response is abrogated by the increased activity of commensal microbiota, as is likely to be the case here, then circulating levels of IL-1β and TNF α, and macrophage activity would remain unaltered following LPS, and microglia would not be activated. We have therefore provided compelling evidence for the significant role of microbiota in the modulation of neuro-immune interactions. Recent investigations using germ-free mice demonstrated that in the absence of gut bacteria, the development and maturation of microglia are impaired, and their response to endotoxin is exacerbated (Erny et al., 2015). The study further demonstrated that a systemic injection of short-chain fatty acids (SCFAs), the products of bacteria-fermented dietary fibres, to germ-free mice, rectified aberrant microglia morphology and function. Given that high concentrations of SCFAs are generated from the microbial metabolism of prebiotics (McCarty and DiNicolantonio, 2015), it is possible that these metabolites contributed to the effects seen in our study.

However, since the expression of SCFA receptors are low or absent in the brain (Erny et al., 2015), it is likely that these molecules have indirect effects on the CNS. For instance, SCFAs release the gut hormone, glucagon-like peptide-1 (GLP-1) (McCarty and DiNicolantonio, 2015), which has both neurotrophic (Savignac et al., 2013) and anti-inflammatory (Dai et al., 2014) actions. Thus, GLP-1 may be a key mediator linking the microbiome to the brain. Another proposed mediator of microbiome-gut-brain axis communication is the vagus nerve, which physically connects the gut and the brain stem. Vagotomy in mice has been reported to mitigate the psychotropic effects of certain probiotics (Bravo et al., 2011). In this instance, the microbiota may stimulate the vagal nerve endings in the intestine via the aforementioned products of microbial metabolism, or even through neurotransmitters produced by the microbes themselves (Cryan and Dinan, 2012). Vagal nerve activation has also been shown to attenuate the effects of central pro-inflammatory cytokines (Garcia-Oscos et al., 2015). Clearly, further research is required to ascertain the mediator(s) of the BGOS effects seen in the current investigation.

We have previously demonstrated an increase of 5-HT2AR expression in the rat cortex, 6 h after an LPS injection (Couch et al., 2013b) and in the mouse 24 h after LPS (Couch et al., 2015). Here we have confirmed that cortical 5-HT2ARs increase with acute inflammation, and have demonstrated that the effect is sustained for 28hrs post-injection. Based on previous findings, we suggest that this elevation of cortical 5-HT2ARs is involved in the central pathophysiological effects of inflammation. Pharmacological evidence supports the role of 5-HT2 receptors in the modulation of anxiety and depression, and non-selective antagonists such as ritanserin and ketanserin are anxiolytic in rodents (Griebel et al., 1997, Nic Dhonnchadha et al., 2003). Furthermore, 5-HT2AR knockout mice are less anxious than controls in the light–dark box test, and the re-introduction of this receptor into the cortex of these mice restores normal anxious behaviour (Weisstaub et al., 2006). These data suggest, therefore, that the LPS-mediated increase of cortical 5-HT2ARs observed in the current study, may underlie the post-inflammatory anxiety displayed in the light–dark box. Mechanistically, LPS-mediated anxious behaviour in rodents has been associated with hyper-excitability of the frontal cortex (Garcia-Oscos et al., 2015). Since the pharmacological stimulation of 5-HT2ARs expressed on pyramidal cells in the prefrontal cortex can increase the local release of glutamate (Mocci et al., 2014), it is reasonable to suggest that the elevation of these receptors contribute to inflammatory cortical hyper-excitability.

Although the mechanisms underlying the modulation of 5-HT2ARs by acute inflammation remain elusive, our data suggest a putative role of IL-1β, since the suppression of this cytokine by BGOS coincides with the normalisation of 5-HT2ARs levels. The mechanism by which BGOS alters cytokine expression in the brain following LPS is likely to be mediated by its impact on peripheral cytokine expression, through the mechanisms discussed above. BGOS has been shown to reduce TNF-induced IL-8 and MIP-3α secretion by the inhibition of an NFkB-dependent pathway (Tzortzis, 2009). The intraperitoneal administration of IL-1β has been shown the induce sickness behaviour in rodents in a manner that is dependent on the synthesis of IL-1β within the CNS (Bluthé et al., 1995), and thus any BGOS-induced reduction in peripheral IL-1β expression, by dampening the host-immune response, would be likely to alter IL-1β expression within the brain.

The interaction between IL-1β and 5-HT2AR has been implied by studies demonstrating that the intracerebroventricular infusion of IL-1β into rats, induces a hyperthermic response that can be blocked by 5-HT2AR antagonists (Chio et al., 2005). However, the molecular basis for this association remains elusive. The current study investigated NMDARs as potential intermediaries of the observed LPS and BGOS effects, since they interact with 5-HT2ARs (Yuen et al., 2008), mediate neurotoxicity in central inflammation (Ma et al., 2014), and are modulated by BGOS (Savignac et al., 2013). In our study, the main effect of LPS was to increase cortical NR2B subunits, which is consistent with earlier observations (Harré et al., 2008, Tanaka et al., 2011), and may have resulted directly from the observed elevation of brain IL-1β (Viviani et al., 2006). If this change enhanced NMDAR function, then it could be reasoned that the elevation of 5-HT2ARs was a homeostatic response to counterbalance NMDAR mediated excitotoxicity. The reduction of IL-1β by BGOS seen in the current study, and by others (Vulevic et al., 2013), may have then attenuated the NMDAR response, and subsequent 5-HT2AR changes. Although further investigations to elucidate the central mechanisms underlying the anxiolytic effect of BGOS post-inflammation are required, there is no doubt that the anti-inflammatory action of this prebiotic is an important factor.

While effects on conditional, ethological test of anxiety (light/dark box) were clearer, our data on the effect of BGOS on LMA during LPS-mediated sickness, were equivocal. The significant three-way interaction between BGOS feeding, LPS injection and time, suggested that the prebiotic influenced the action of LPS on LMA. The reduced LMA in Water/LPS compared to Water/Sal was not apparent in corresponding BGOS-fed animals, and on face, might be interpreted as an attenuation of the LPS effect by BGOS. However, although prior exposure to a BGOS diet influenced subsequent LMA data, it was not simply the case that BGOS protected against LPS-induced hypoactivity. It was also clear from the current study that BGOS supplementation did not affect LPS-mediated reductions in marble burying behaviour. One interpretation is that the prebiotic only influences anxiety-related behaviours which involve an approach/avoidance conflict, and not other behaviours potentially related to emotionality, such as marble burying, but this would require further investigation.

Overall, the anxiolytic and anti-inflammatory effects of BGOS in the current study were in-keeping with the actions of certain probiotics that reduce inflammation or anxiety (Bercik et al., 2010, Cryan and Dinan, 2012, Wang and Kasper, 2014). However, BGOS only had a discreet behavioural effect in the animals that did not receive LPS (cf. LMA of BGOS/SAL vs Water/SAL at 70 and 80 min). This was surprising in view of our recent data showing that BGOS administration to healthy human volunteers, improved emotional processing (Schmidt et al., 2015), and that *Bifidobacteria* probiotics have anxiolytic effects in healthy animals (for review see Cryan and Dinan, 2012). The latter discrepancy may arise from the different mouse strains used in each study. The mice in the probiotic study are known to be innately anxious and display greater anxiety than other mouse strains (Jacobson and Cryan, 2005, Savignac et al., 2011, Savignac et al., 2014, Ohland et al., 2013). Thus, a pre-existing high basal level of anxiety may be required to reveal the anxiolytic action of pre- and probiotics.

## 5 Conclusions

We have demonstrated that dietary supplementation with the prebiotic BGOS, attenuated anxiety, but not marble burying behaviour, in mice receiving a single injection of LPS. The beneficial effects of BGOS on behaviour were most prominent in the late-stage response to LPS, which has been likened to post-infection fatigue (Couch et al., 2015). The prebiotic also prevented the LPS-mediated increase in IL-1β and 5-HT2AR expression in the frontal cortex, in the absence of altered 5-HT metabolism. Our study has revealed a more direct link between the IL-1β and altered 5-HT2AR receptor expression than previously envisaged. Moreover, in spite of its molecular links with both IL-1β and 5-HT2AR, the NMDAR did not appear to be involved in the combined effects of LPS and BGOS in the current study. We therefore propose that the anxiolytic action of BGOS on LPS injected mice was the result of the prebiotic’s anti-inflammatory properties, which suppressed the increase of IL-1β, and subsequent elevation of 5-HT2ARs that may underlie post-inflammatory anxiety behaviour. Our results therefore provide further compelling evidence for the central benefits of BGOS supplementation.

## Figures and Tables

**Table 1:** Cortical 5-HT and 5-HIAA concentrations following the oral administration of water or BGOS and a single injection of saline or LPS. Data are expressed as means ± SEM. The number of mice/group are indicated in parentheses.

	[nmol/g Tissue]			
	Water/SAL	Water/LPS	BGOS/SAL	BGOS/LPS
	(*n* = 12)	(*n* = 11)	(*n* = 12)	(*n* = 11)
5-HT:	2.17 ± 0.39	0.99 ± 0.11	1.95 ± 0.46	1.49 ± 0.33
5-HIAA:	0.76 ± 0.16	0.39 ± 0.04	0.95 ± 0.13	1.08 ± 0.30
5-HIAA/5-HT:	0.35 ± 0.05	0.31 ± 0.06	0.38 ± 0.08	0.52 ± 0.10
